# Comparative transcriptome analysis of hypothalamus-regulated feed intake induced by exogenous visfatin in chicks

**DOI:** 10.1186/s12864-018-4644-7

**Published:** 2018-04-11

**Authors:** Zhuanjian Li, Xuelian Liu, Panpan Zhang, Ruili Han, Guirong Sun, Ruirui Jiang, Yanbin Wang, Xiaojun Liu, Wenya Li, Xiangtao Kang, Yadong Tian

**Affiliations:** College of Animal Science and Veterinary Medicine, Henan Agricultural University, Zhengzhou, 450002 China

**Keywords:** Visfatin, Feed intake, Hypothalamus, Transcriptomics, DEGs

## Abstract

**Background:**

The intracerebroventricular injection of visfatin increases feed intake. However, little is known about the molecular mechanism in chicks. This study was conducted to assess the effect of visfatin on the feeding behavior of chicks and the associated molecular mechanism.

**Results:**

In response to the intraventricular injection of 40 ng and 400 ng visfatin, feed intake in chicks was significantly increased, and the concentrations of glucose, insulin, TG, HDL and LDL were significantly altered. Using RNA-seq, we identified DEGs in the chick hypothalamus at 60 min after injection with various doses of visfatin. In total, 325, 85 and 519 DEGs were identified in the treated chick hypothalamus in the LT vs C, HT vs C and LT vs HT comparisons, respectively. The changes in the expression profiles of DEGs, GO functional categories, KEGG pathways, and PPI networks by visfatin-mediated regulation of feed intake were analyzed. The DEGs were grouped into 8 clusters based on their expression patterns via K-mean clustering; there were 14 appetite-related DEGs enriched in the hormone activity GO term. The neuroactive ligand-receptor interaction pathway was the key pathway affected by visfatin. The PPI analysis of DEGs showed that POMC was a hub gene that interacted with the maximum number of nodes and ingestion-related pathways, including POMC, CRH, AgRP, NPY, TRH, VIP, NPYL, CGA and TSHB.

**Conclusion:**

These common DEGs were enriched in the hormone activity GO term and the neuroactive ligand-receptor interaction pathway. Therefore, visfatin causes hyperphagia via the POMC/CRH and NPY/AgRP signaling pathways. These results provide valuable information about the molecular mechanisms of the regulation of food intake by visfatin.

**Electronic supplementary material:**

The online version of this article (10.1186/s12864-018-4644-7) contains supplementary material, which is available to authorized users.

## Background

Poultry feed intake is one of the important factors restricting production efficiency. Feed intake is closely related to animal health and production efficiency. For chicks, there is a close relationship between weight gain and feed intake; strong pecking desire is the premise to improve feed intake in chicks [1, 2]. The feeding response in poultry is a complex, large and elaborate network that includes various hormones, neurotransmitters, secreted factors and cytokines [3]. The regulation of the feeding behavior in poultry is mainly controlled by the peripheral and central nervous system. Peripheral tissues sense nutrition metabolism in the body through sensors and regulate appetite by secreting a variety of peptides and steroid hormones [4]. The central nervous system plays an important role in the energy homeostasis and feeding behavior in mammals and birds, and a complex neuronal network within the hypothalamus regulates appetite and energy balance [5, 6]. As the control center, the hypothalamus can regulate feed intake by the synthetic action of various feeding-promotion and feeding-suppression neuropeptides. The structures and functions of numerous hypothalamic neuropeptides, such as neuropeptide Y (NPY), agouti-related protein (AgRP), orexin (ORX), growth hormone-releasing hormone (GHRH), galanin (GAL), melanin-concentrating hormone (MCH), proopiomelanocortin (POMC), galanin-like peptide (GALP) and corticotropin releasing factor (CRF), have been widely studied and confirmed [7–11]. Peripheral neuropeptides, such as cholecystokinin (CCK), ghrelin, and peptide YY (PYY) can send feedback signals to the central nervous system by regulating the functions of the gastrointestinal tract, including movement, secretion and absorption, thus playing a role in appetite regulation [12]. In addition, after being stimulated by nutrients and environmental factors, central and peripheral tissues produce a variety of signaling molecules, such as neuropeptides, hormones and metabolic products, that can bind to specific receptors and initiate signaling cascades in specific neuron subgroups in the hypothalamus, thereby playing a physiological role in the regulation of poultry feed intake and energy expenditure at both the cellular and systemic levels [13].

Visfatin is a newly discovered adipocytokine that plays an important role in inflammation, insulin resistance, cardiovascular disease, apoptosis and tumor formation. As a secreted cytokine, visfatin can also induce the expression of a variety of inflammation-associated factors, such as PI3K, NF-κB1, TNF-α, IL-1β and IL-6 [14–17]. Studies have described the up-regulation of visfatin in several immune cells, including monocytes, macrophages, dendritic cells, and lymphocytes [18, 19]. Visfatin, with an insulin-like function of reduction of blood glucose and promotion of adipocyte differentiation, can mediate the regulation of glucolipid metabolism [17, 20–22] Until now, studies on visfatin have mainly focused on humans and mammals, while very few have investigated the role of visfatin in birds. Visfatin is expressed in multiple tissues in chicks [23]. In turkeys, visfatin is expressed not only in the plasma and peripheral tissues but also in ovarian cells [24]. In broiler chickens, the expression of visfatin in muscles is higher than in fat tissues [25, 26]. In addition, one study showed that changes of visfatin expression in testis tissue can affect sexual maturity in chickens by affecting spermatogenesis and steroidogenesis [27]. In a preliminary study, we explored the expression of visfatin in tissues of AA broilers and silkies during their development and detected a significant correlation between genetic variation in this gene and early growth traits of chicks [28].

There are few reports on visfatin as an appetite regulatory factor. Cline et al. suggested in their study that the intracerebroventricular (ICV) injection of human recombinant visfatin can significantly increase the appetite of broiler chicks [29]; this conclusion was further validated in mice. Brunetti et al. speculated that this increase may be caused by a decrease in the activity of dopamine (DA), CART and corticotropin releasing hormone (CRH) in the hypothalamus [30, 31]. These results suggest that visfatin can be used as a potential feeding-promotion factor, but the mechanism is not clear. Therefore, to evaluate the effects of visfatin on food intake in chicks, we administered visfatin through ICV injection. With Roman Brown chicks as the research model, different doses of visfatin were administered via trace stereotactic ICV injection to observe the effect on feed intake. Then, using Illumina high-throughput sequencing, the hypothalami from these chicks were analyzed via transcriptome profiling to deeply explore the related genes affected. In addition, we analyzed the corresponding gene ontology (GO) and signaling pathways to understand the molecular regulation of visfatin in poultry feeding.

## Results

### Effect of the ICV injection of visfatin on feed intake in chicks

**Fig. 1:**
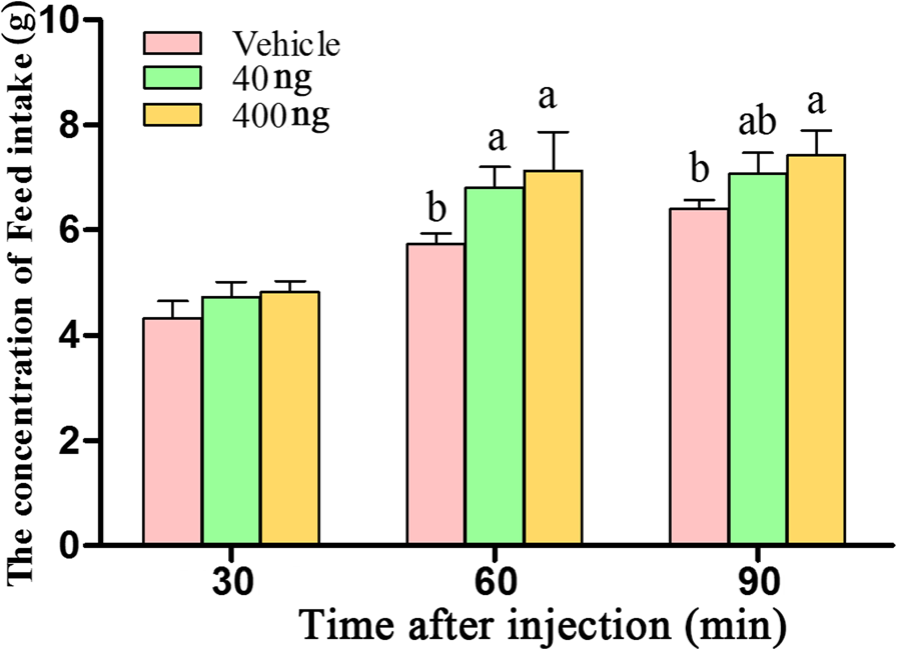
Regulation of food intake by the intracerebroventricular injection of visfatin in neonatal of chicks. Data are presented as the mean ± SE (*n* = 9). Different letters (a and b) represent statistically significant differences among the vehicle, LT and HT groups (*P* < 0.05), analyzed using SSR

The ICV injection of visfatin increased feed intake in chicks. Both 40 ng and 400 ng of visfatin affected feed intake at 30, 60 and 90 min post-injection; however, feed intake was not significantly increased at 30 min post-injection. The cumulative feed intake of chicks in the LT and HT groups was significantly increased compared with that of the chicks in the C group (*P* < 0.05). However, there was no significant difference between the LT and HT groups in the cumulative feed intake (*P* > 0.05) at 60 min post-injection. Feed intake was only significantly increased by 400 ng of visfatin at 90 min post-injection (Fig. 1). Thus, the ICV injection of visfatin can significantly improve feed intake in chicks.

### Influence of ICV visfatin on relative plasma hormone and metabolite levels

**Fig. 2:**
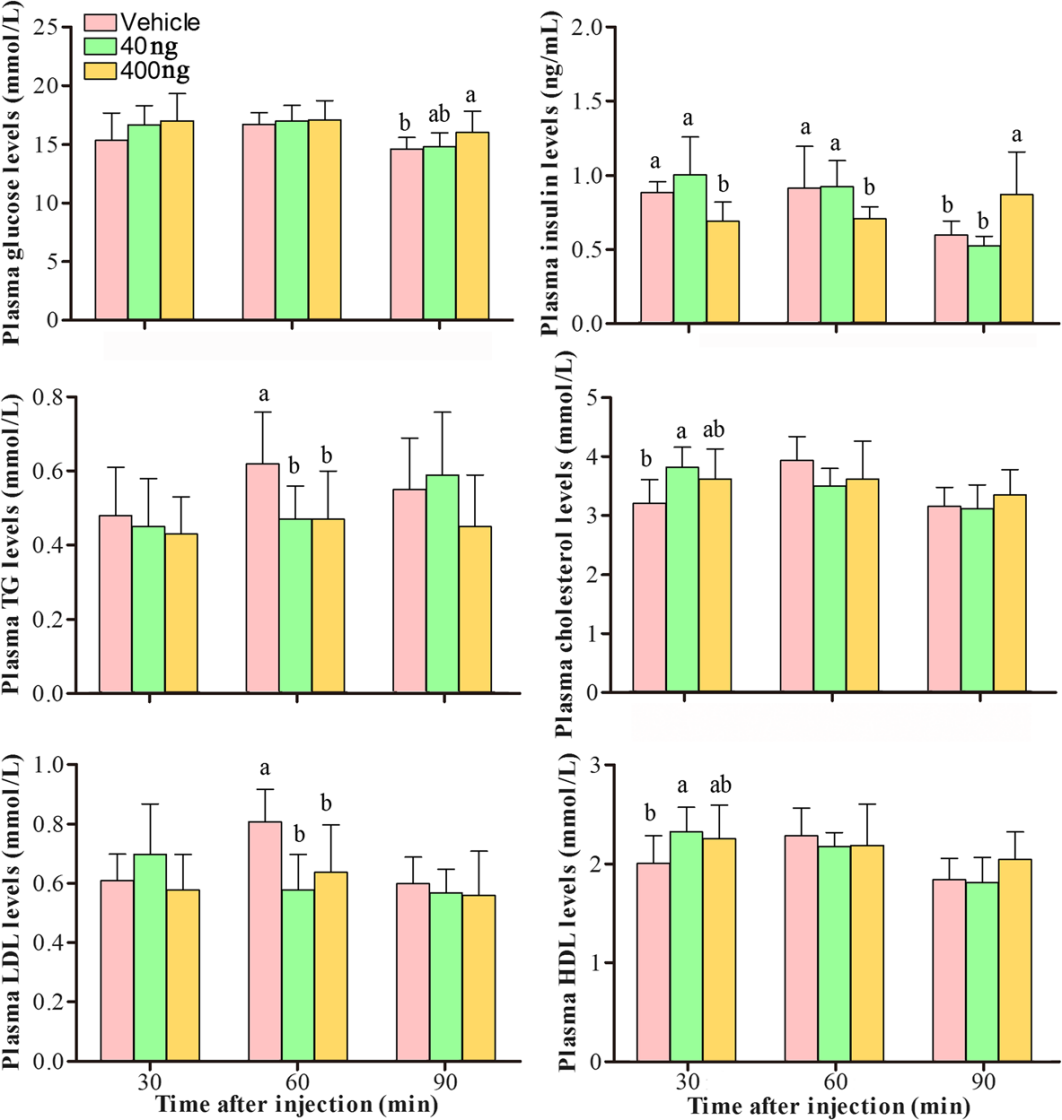
Effects of visfatin treatment on plasma hormone and metabolite levels. Data are presented as the mean ± SE (n = 9). Different letters (a and b) represent statistically significant differences among the vehicle, LT and HT groups (*P* < 0.05), analyzed using SSR

Compared with the effect of the vehicle, plasma glucose concentration was significantly increased by 400 ng visfatin at 90 min post-injection, and although the plasma glucose concentration was increased by 40 ng and 400 ng of visfatin at 30 and 60 min post-injection, the results were not significant (*P* > 0.05). The serum insulin concentration was decreased at 30 and 60 min post-injection with the increase in visfatin dose. The insulin concentration was highest in the LT group, and it was significantly lower in the HT group than in the LT and C groups. The insulin concentration in the HT group was significantly higher than that of the LT and C groups at 90 min post-injection. The serum TG and LDL levels were not significantly changed at 30 and 90 min post-injection. Serum TG, HDL and LDL were all markedly decreased at 60 min post-injection (Fig. 2). Therefore, visfatin can promote feed intake in Roman Brown cockerels and participate in the regulation of serum saccharides and lipids.

### Illumina sequencing and analysis of mRNA expression

To identify genes involved in visfatin-induced intake at the transcriptional level in chicks, nine cDNA preparations were sequenced from the C, LT and HT groups, with three biological replicates in each group. A total of 554,961,344 raw reads and 540,174,404 clean reads were obtained from the 9 cDNA libraries. The proportion of clean reads rate over the raw reads in the 9 libraries ranged from 97.19% to 97.52%. Overall, 81.87% to 82.99% of the clean reads were aligned against the *Gallus gallus* reference genome, and the uniquely mapped reads ranged from 81.87% to 82.99%. Among these mapped reads, 6.3% to 7.7% were mapped to introns, 60.3% to 63.7% were mapped to exons, and 29.5% to 33.1% were mapped to intergenic regions. The average GC content of the clean reads from the 9 libraries was 48.66%. The proportion of reads with the phred quality value greater than 20 among the clean reads ranged from 94.87% to 97.52%, and the Q30 among the clean reads ranged from 87.0% to 91.7% (Table 1). The error rate of single base ranged from 0.03% to 0.04%. The *R*^*2*^ value between each repeated sample was greater than 0.96, which was higher than the required *R*^*2*^ of 0.92 under ideal sampling and experimental conditions (Additional file 1: Figure S1). Therefore, the quality of the sequencing results was excellent, with good repeatability among the samples and reasonable and reliable sample selection.

**Table 1:** Analyses of clean reads in the chicken hypothalamus

Sample name	Raw reads	Clean reads	Mapping rate	Uniquely mapped rate	Exon%	Intron%	Intergenic%	GC content %	Average RPKM
C1	63,306,944	61,717,348	82.01%	81.11%	60.3	6.6	33.1	49.10	21.17
C2	60,243,690	58,704,022	82.29%	81.43%	60.6	7.1	32.3	48.43	20.97
C3	62,219,776	60,616,596	82.35%	81.45%	61.1	7.1	31.8	48.58	20.79
HT1	68,168,766	66,327,478	82.3%	81.41%	60.5	7.7	31.8	48.34	21.07
HT2	61,505,548	59,694,092	82.29%	81.4%	60.8	7.6	31.6	48.50	21.16
HT3	57,359,412	55,863,346	82.46%	81.58%	61.3	7.2	31.5	48.69	21.28
LT1	60,860,822	59,147,790	81.87%	80.99%	62.2	7.5	30.2	48.88	20.94
LT2	59,589,616	57,924,762	82.99%	82.12%	63.3	6.3	30.4	48.44	21.24
LT3	61,706,770	60,178,970	82.93%	82.06%	63.7	6.8	29.5	48.51	21.28

### DEGs induced by visfatin in the hypothalamus regulate feed intake

**Fig. 3:**
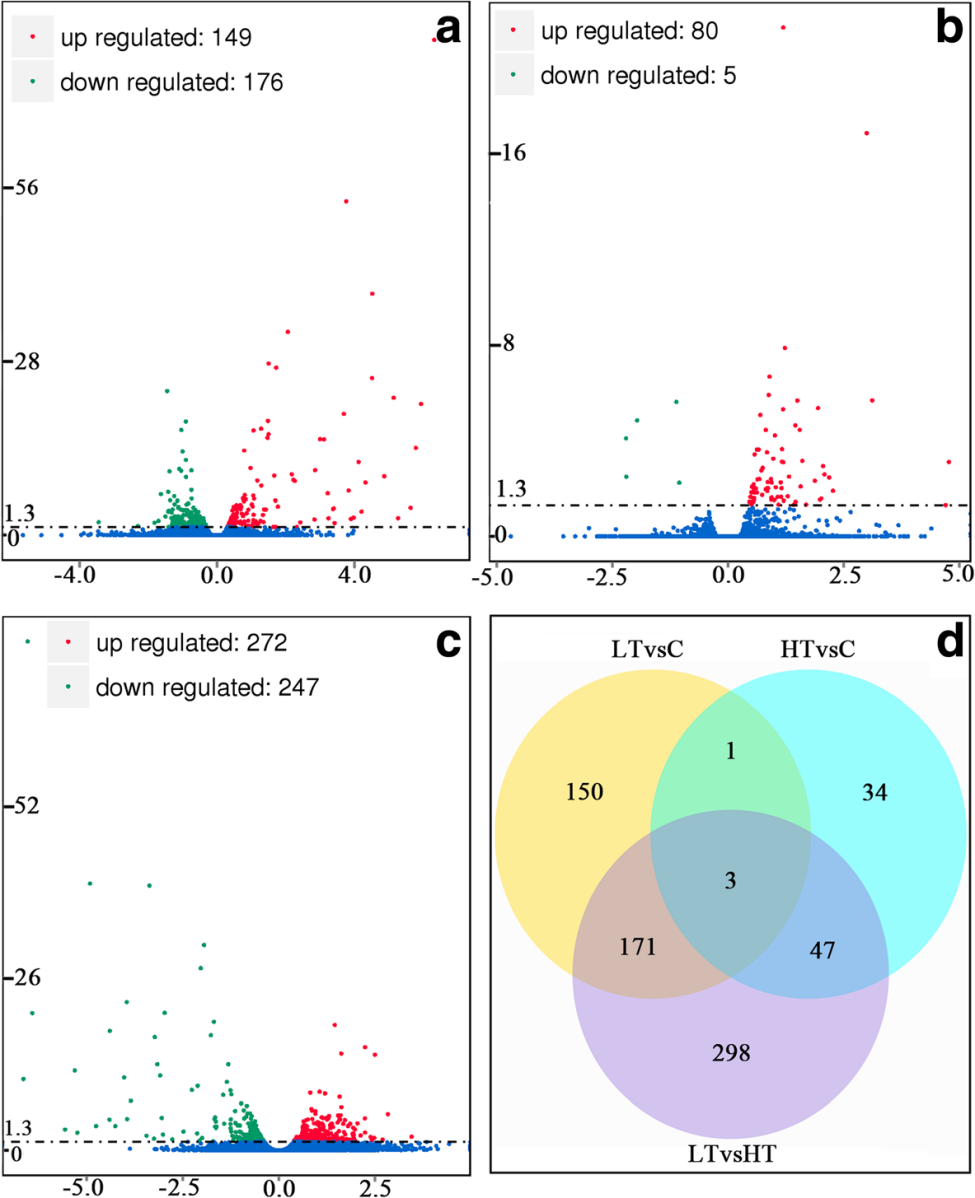
Analyses of DEGs in three comparisons. **a**, **b** and **c** represent LT vs C, HT vs C and LT vs HT groups, respectively. Significant DEGs are shown as red (up) or green (down) dots. The lack of significant difference between gene expression is indicated by blue dots. Ordinates represent the magnitude of gene expression changes. The x-axis represents the value of log2(fold-change), and the y-axis shows the value of -log10(padj). **d** Venn diagram showing the overlap of the DEGs in three comparisons. DEGs that are common to multiple time points are shown by the overlap

The sequencing results of three experimental groups were compared with the reference gene group, and 28,100 genes were identified, including 17,108 annotated genes and 10,992 unknown genes. For experiments with biological repeats, since DESeq eliminated biological variation, our screening standard for the differential gene was adjusted to *P* < 0.05. In total, 704 differentially expressed mRNAs were obtained by pairwise comparisons (LT vs C, HT vs C and HT vs LT) of samples collected from the three groups (Fig. 3, Additional file 2: Table S2). There were a total of 325 DEGs between the LT and C groups, including 149 up-regulated and 176 down-regulated genes (Fig. 3a), and a total of 85 DEGs between the C group and HT group, including 80 up-regulated and 5 down-regulated genes (Fig. 3b). There were 519 DEGs between the LT group and the HT group, including 272 up-regulated and 247 down-regulated genes (Fig. 3c). Additionally, 174 DEGs were common between groups LT vs C and HT vs LT; 50 DEGs were common between groups HT vs C and HT vs LT; 4 DEGs were common between groups LT vs C and HT vs C groups (Fig. 3d), including *thyroid hormone* (*TH*), *GHRH*, and two novel genes (Novel10261 and Novel09279); and 3 DEGs were common between groups LT vs C, HT vs C and HT vs LT groups, including *TH* and two novel genes.

### Series-cluster analysis

**Fig. 4:**
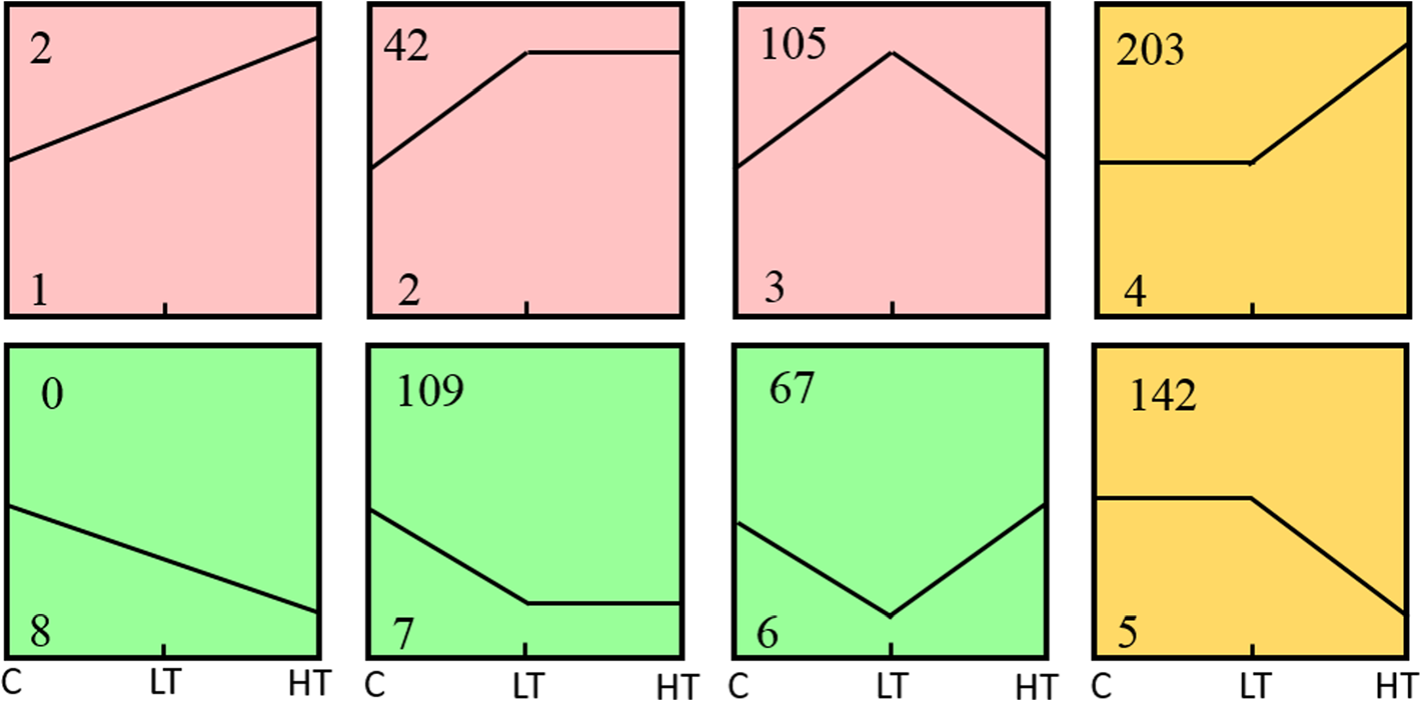
Eight clusters of genes with unique patterns of expression in the three groups. Clusters were ordered based on the number of genes assigned to them. The cluster number is shown in the bottom left corner of each cluster square. The number of genes grouped in each cluster is shown in the top left corner of each cluster square. The distances from the left-most point, the middle point, and the right-most point of the polyline within each cluster square to the lower line of each square represent the relative (unscaled) gene expression levels among the C, LT, and HT groups

Based on the trend in expression, series-cluster analysis of these DEGs classified them into eight clusters among the three groups. However, the genes were only significantly enriched in seven clusters: 2 DEGs in cluster 1; 42 DEGs in cluster 2; 105 DEGs in cluster 3; 203 DEGs in cluster 4; 142 DEGs in cluster 5; 67 DEGs in cluster 6; and 109 DEGs in cluster 7 (Fig. 4).

**Fig. 5:**
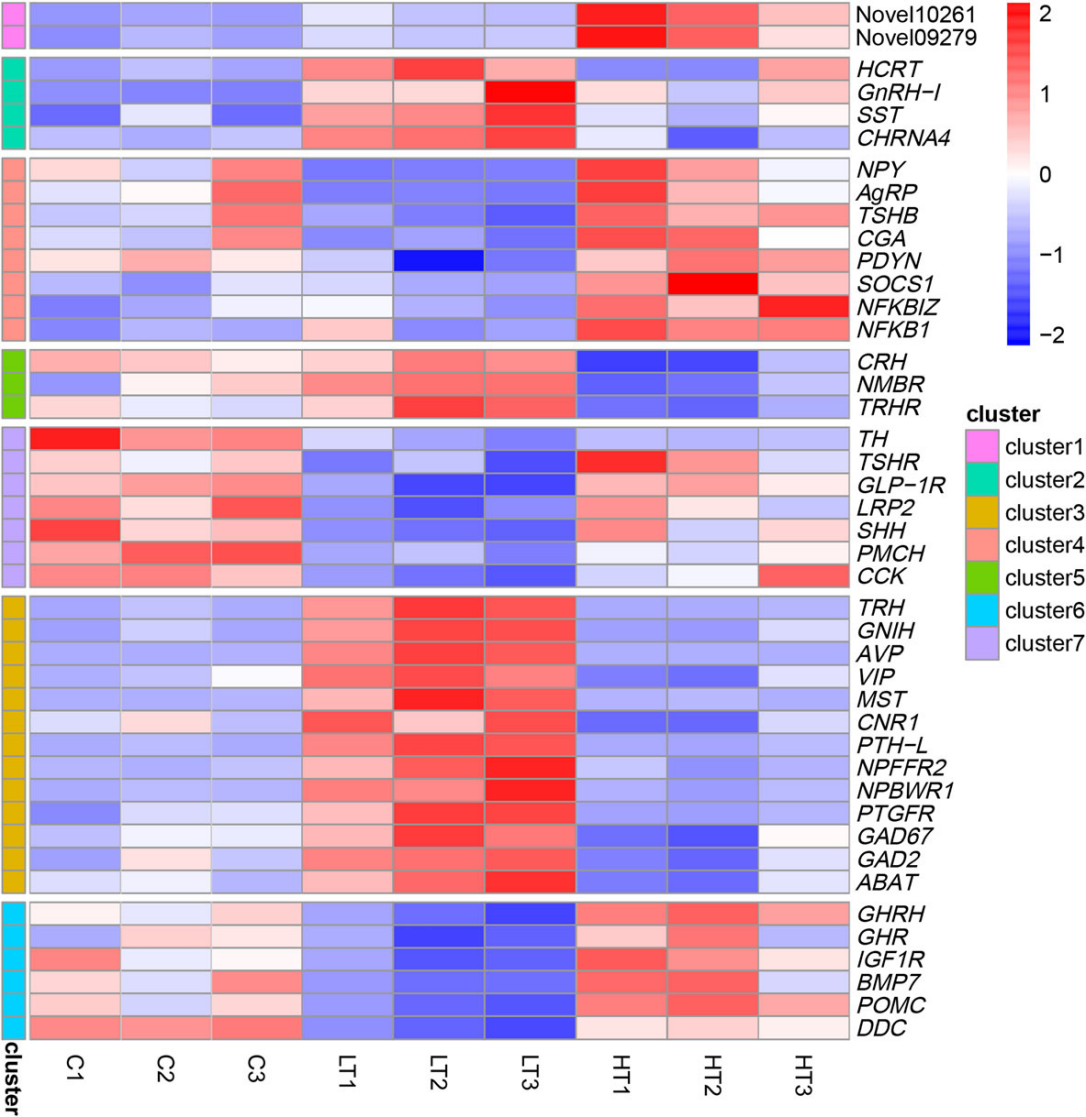
Heatmap of candidate ingestion-related DEGs

Among them, cluster 1 contained 2 DEGs (Novel10261 and Novel09279) whose expression was increased in all three groups. Cluster 2 contained ingestion-related DEGs, including *hypocretin neuropeptide precursor* (*HCRT*), gonadotropin-releasing hormone I (GnRH-I), somatostatin (SST) and cholinergic receptor nicotinic alpha 4 subunit (CHRNA4), whose expression increased in the LT group compared to the level in the C group, with a non-significant increase in HT. Cluster 4 contained ingestion-related DEGs, including *NPY*, AgRP, thyroid-stimulating hormone beta (TSHB), common glycoprotein alpha chain (CGA), prodynorphin (PDYN), suppressor of cytokine signaling 1 (SOCS1), nuclear factor kappa B inhibitor zeta (NFKBIZ) and nuclear factor kappa B subunit 1 (NFKB1). The change in gene expression among these three groups indicated that these genes may play a positive role in the hypothalamus in regulating feed intake. Cluster 5 contained ingestion-related DEGs, including *CRH, CRF*, neuromedin B receptor (NMBR) and thyrotropin-releasing hormone receptor (TRHR). Cluster 7 contained ingestion-related DEGs, including *TH*, thyroid-stimulating hormone receptor (TSHR), glucagon-like peptide 1 receptor (GLP-1R), LDL receptor-related protein 2 (LRP2), sonic hedgehog (SHH), pre-pro melanin-concentrating hormone (PMCH) and the peptide hormone CCK. The change in gene expression among these three groups indicated that these genes may play a negative role in the hypothalamus in regulating feed intake. The genes in clusters 4 and 5 are induced by a high dose of visfatin but not by a low dose. Cluster 3 contained ingestion-related DEGs, including *thyrotropin-releasing hormone* (*TRH*), gonadotropin-inhibitory hormone (GnIH), arginine vasopressin (AVP), vasoactive intestinal peptide (VIP), mesotocin-neurophysin I (MST), cannabinoid receptor 1 (CNR1), parathyroid hormone-like (PTH-L), neuropeptide FF receptor 2 (NPFFR2), neuropeptides B/W receptor 1 (NPBWR1), prostaglandin F receptor (PTGFR), glutamate decarboxylase 1, 67 kDa (GAD67), glutamate decarboxylase 2, 67 kDa (GAD2) and 4-aminobutyrate aminotransferase (ABAT), whose expression increased in the LT group compared to that in the C group and significantly decreased in the HT group compared to that in the LT group. Cluster 6 contained ingestion-related DEGs, including *GHRH*, growth hormone receptor (GHR), insulin-like growth factor 1 receptor (IGF1R), bone morphogenetic protein 7 (BMP7) and POMC, whose expression decreased in the LT group compared to that in the C groups but significantly increased in the HT group compared to that in the LT group (Fig. 5). Therefore, these are likely candidate genes that play an important role in the hypothalamus in regulating feed intake induced by visfatin in chicks.

Moreover, there were some inflammation- and immune-related DEGs, including interleukin (IL)-8, IL-13, IL-22, IL-34, NFKB1, NFKB inhibitor alpha (NFKBIA), CD83, CD44 and phosphoinositide 3-kinase regulatory subunit 5 (PIK3R5).

### GO enrichment analysis

**Fig. 6:**
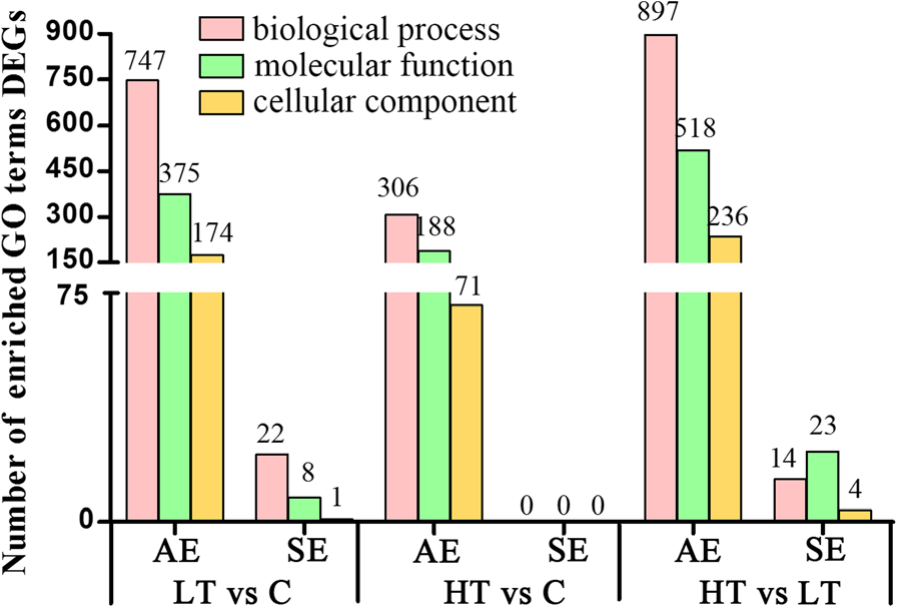
GO enrichment of DEGs in different comparative groups. AE and SE represent all enrichment and significant enrichment, respectively

We employed GOseq to compare the GO classifications of the groups of up-regulated and down-regulated genes (adjusted *P* < 0.05) [32]. To evaluate the differential GOs in the three groups, the significantly up- and down-regulated genes were compared between the C and LT, C and HT and LT and HT groups. Overall, 704 DEGs were enriched in 1893 GO terms; 448 GOs were common among groups LT and C, groups HT and C and groups HT and LT. Among them, 231 GOs were enriched in biological processes (BP), 151 were enriched in molecular function (MF), and 66 were enriched n cellular component (CC). In total, 31 GOs were significantly different between the LT and C groups; among them, 22 GOs were enriched in BP, 8 were enriched in MF, and 1 was enriched in CC. Moreover, 41 GOs were significantly different between the LT and HT groups; among them, 14 GOs were enriched in BP, 23 were enriched in MF, and 4 were enriched in CC. There were no significantly different GOs between the HT and C groups (Fig. 6, Additional file 3: Table S3). The top 30 significantly enriched GO terms are shown in Additional file 4: Figure S2. The most significantly enriched GO term between the C and LT groups was hormone activity. The top three GO terms were nucleic acid-binding transcription factor activity, sequence-specific DNA-binding transcription factor activity, and hormone activity between the LT and C groups; G-protein coupled receptor binding, chemokine activity, and chemokine receptor binding between the HT and C groups; and nucleic acid-binding-transcription factor activity, sequence-specific DNA-binding transcription factor activity, and biological regulation between the HT and LT groups.

There were 14 DEGs enriched in the hormone activity GO term, including 8 up-regulated genes (SST, PTH-L, VIP, TRH, MST, GnRH-I, GnIH and AVP) and 6 down-regulated genes (Natriuretic peptide C type 3 (CNP3), PMCH, POMC, CCK, ATP-binding cassette subfamily A member 4 (ABCA4) and GHRH). Moreover, there were some ingestion-related GO terms, including orexigenic neuropeptide QRFP receptor binding, neuropeptide signaling pathway, neuropeptide hormone activity, hormone-mediated signaling pathway, cellular response to hormone stimulus, feeding behavior and neurohypophyseal hormone activity.

Additionally, there were 36 DEGs enriched the transmembrane signaling receptor activity GO term, including ingestion-related genes such as TRHR, NPFFR2, GRM5, NPBWR1, CNR1, PTGFR and NMBR, between the HT and LT groups. There were 6 DEGs enriched in the immune response and immune system process GO terms, including CCL4, IL-8 L1, TNFSF15, and IL-8, between the HT and C groups.

### KEGG pathway enrichment analysis

To further determine the biological pathways that were significantly (adjusted *P* < 0.05) modulated by visfatin, KEGG pathway enrichment was performed using KOBAS [33]. The top 20 significantly enriched pathways for the LT vs C, HT vs C, and LT vs HT comparisons are shown in Additional file 5: Figure S3. There were 59 DEGs enriched in 49 pathways between the LT and C groups. Among them, 28 DEGs were significantly enriched in 9 pathways. There were 21 DEGs enriched in 35 pathways between the HT and C groups. Among them, 11 DEGs were significantly enriched in 7 pathways. There were 124 DEGs enriched in 85 pathways between the HT and C groups. Among them, 38 DEGs were significantly enriched in 3 pathways (Additional file 6: Table S4). The ECM-receptor interaction pathway, the neuroactive ligand-receptor interaction pathway, and the alanine, aspartate and glutamate metabolism pathway were the top three pathways for the LT vs C comparison. The toll-like receptor signaling pathway, the NOD-like receptor signaling pathway, and the cytokine-cytokine receptor interaction pathway were the top three pathways for the HT vs C comparison. The neuroactive ligand-receptor interaction pathway, the ECM-receptor interaction pathway, and the cytokine-cytokine receptor interaction pathways were the top three pathways for the HT vs LT comparison.

**Fig. 7:**
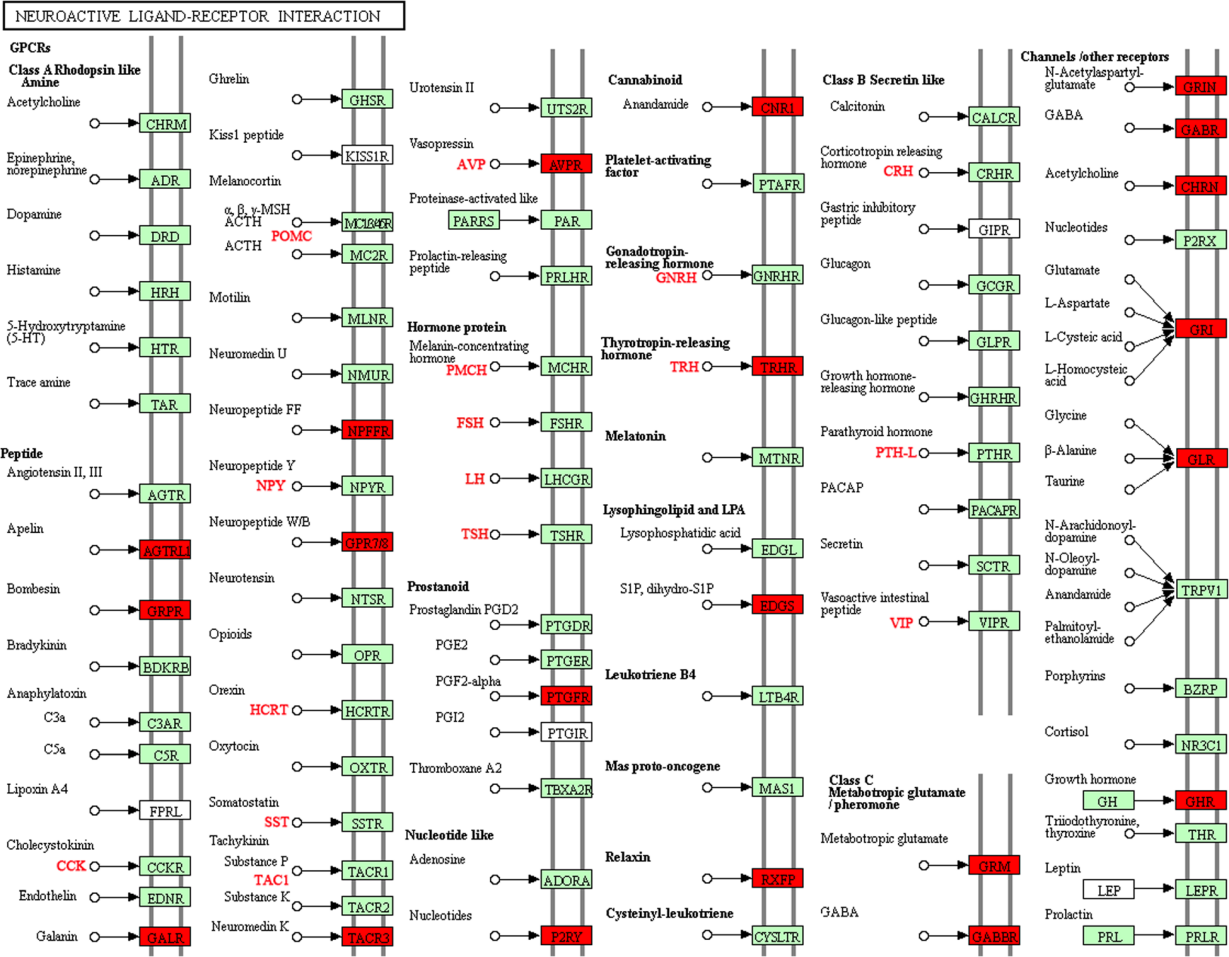
DEGs involved in the neuroactive ligand-receptor interaction pathway. Red box plot shows receptor genes, and the red font shows ligand-encoding genes in the pathway

Interestingly, we found that the neuroactive ligand-receptor interaction pathway and the ECM-receptor interaction pathway were common between the LT vs C and HT vs LT comparisons. We found that 38 DEGs were enriched in the neuroactive ligand-receptor interaction pathway for the HT vs C comparisons (Fig. 7). Further analysis indicated that the 38 DEGs were enriched in genes that were appetite-related and involved in the neuroactive ligand-receptor interaction pathway, including POMC, CRH, NPY, TRH, VIP, CGA, TSHB, TH, GHRH, CCK, HCRT, SST, ABAT, *NPFFR2* and *GLP-1R*. The Pearson’s correlation coefficient between the expression of receptor genes and ligand genes in the neuroactive ligand-receptor interaction pathway was 0.63 (Additional file 7: Table S5). The neuroactive ligand-receptor interaction pathway includes all receptors and ligands on the plasmalemma that are related to intracellular and extracellular signaling pathways [34], comprising a total of 261 genes. This pathway includes many appetite-related genes. Thus, this pathway plays a very important role in feeding regulation after ICV injection of visfatin in chicks. Moreover, the melanogenesis pathway was common between the LT vs C and HT vs LT comparisons. POMC is an important gene in this pathway, which plays an important role in the regulation of feed intake. Furthermore, we found that some pathways such as adipocytokine signaling pathway, insulin signaling pathway and melanogenesis pathway are involved in the energy homeostasis and regulation of feed intake in chicks, and genes including *SOCS1*, *NFKB1*, *NFKBIA* and *PIK3R5* are also related to feed intake regulation.

A previous study suggested that AMPK activity in the hypothalamus is modulated by fasting and feeding [35]. AMPK may regulate feeding behavior through the AMPK-ACC-CPT1-NPY and AMPK-mTOR-p70s6k/4EBP1-NPY pathways [36]. However, the expression of the relevant genes, including AMPK, acetyl-CoA carboxylase (ACC; a lipogenic enzyme), carnitine palmitoyltransferase-1 (CPT1), long-chain acyl-CoAs (ACSL1), mammalian target of rapamycin (mTOR; a serine-threonine kinase), tuberous sclerosis complex (TSC), p70 ribosomal S6 kinase 1 (P70s6k) and the translation initiation factor 4E-binding protein (4EBP1), were not significantly different among the three groups in our study (Table 2). Therefore, visfatin regulates feeding behavior by other pathways than the AMPK-ACC-CPT1-NPY and AMPK-mTOR-p70s6k/4EBP1-NPY pathways.

**Table 2:** The RPKM and adjusted P-value of gene in the AMPK pathway

Gene symbol	C	LT	HT	LT vs C	HT vs C	HT vs LT
	RPKM	RPKM	RPKM	p-adj	p-adj	p-adj
*AMPK*	13.56	13.68	13.21	1.00	1.00	0.97
*ACC*	24.08	23.00	21.18	1.00	1.00	0.75
*CPT1*	18.81	19.67	19.92	1.00	1.00	1.00
*CPT2*	13.55	13.24	12.07	1.00	1.00	0.83
*ACSL3*	21.50	21.76	19.64	1.00	1.00	0.69
*AMPK*	13.56	13.68	13.21	1.00	1.00	0.97
*mTOR*	18.66	18.26	17.05	1.00	1.00	0.82
*TSC1*	20.38	19.86	19.35	1.00	1.00	0.98
*TSC2*	14.42	13.58	13.48	1.00	1.00	1.00
*P70s6k*	11.57	10.55	11.86	1.00	1.00	0.90
*4EPB1*	63.19	60.58	61.72	1.00	1.00	1.00

### PPI network analysis

**Fig. 8:**
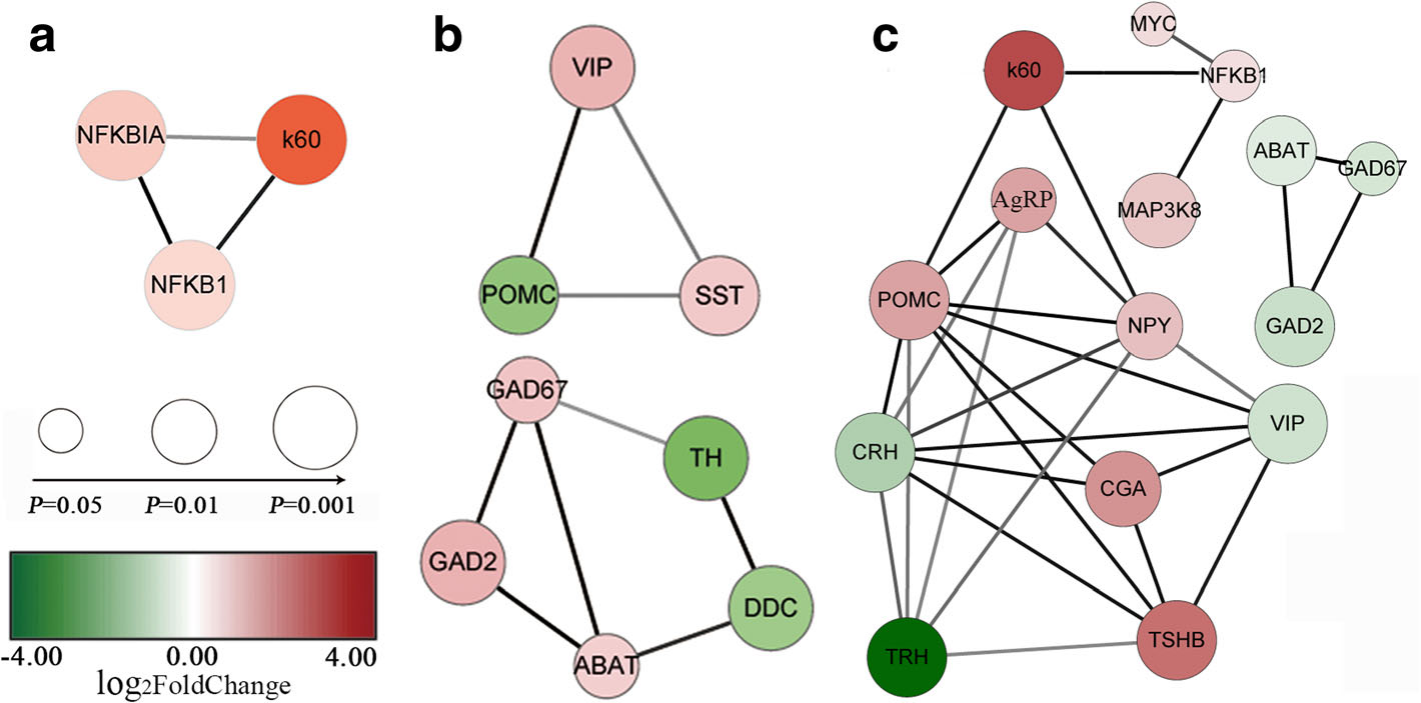
PPI network of DEGs. **a**, **b** and **c** represent HT vs C, LT vs C and LT vs HT, respectively. The color of nodes indicates the degree of fold-change. Red nodes represent up-regulated DEGs and green nodes represent down-regulated DEGs. The node size represents -logP; a smaller *P*-value indicates a larger node size. Lines between DEGs represent interactions between them

The PPI network was constructed using the extracted target gene list from the STRING database (https://string-db.org, Organism: *Gallus gallus*). The figure shows that POMC was a hub gene that interacted with the maximum number of nodes. The DEGs from the LT vs C PPI network contained 15 nodes and 12 edges, including 7 up-regulated genes and 8 down-regulated genes. Among them, there was an ingestion-related pathway that had SST, POMC, NPY, VIP and TH (Fig. 8b). The DEGs from the HT vs C PPI network contained 6 nodes and 5 edges, including 7 up-regulated genes. Among them, there was an inflammation-related pathway that had K60, NFKB1 and NFKB1A (Fig. 8a). The DEGs from the GT vs LT PPI network contained 45 nodes and 41 edges, including 7 up-regulated genes and 8 down-regulated genes. Among them, there was an inflammation-related pathway that had IL-8, K60, NFKB1 and NFKB1A and an ingestion-related pathway including NPY, AgRP, POMC, CRH, TRH, VIP, CGA and TSHB (Fig. 8c). Interestingly, we found that a pathway was common between the LT vs C and HT vs LT comparisons, with ABAT, GAD67 and GAD2 (Fig. 8). These genes were enriched in the KEGG pathways alanine, aspartate and glutamate metabolism, butanoate metabolism and beta-alanine metabolism.

### Validation of gene expression using qRT-PCR

**Fig. 9:**
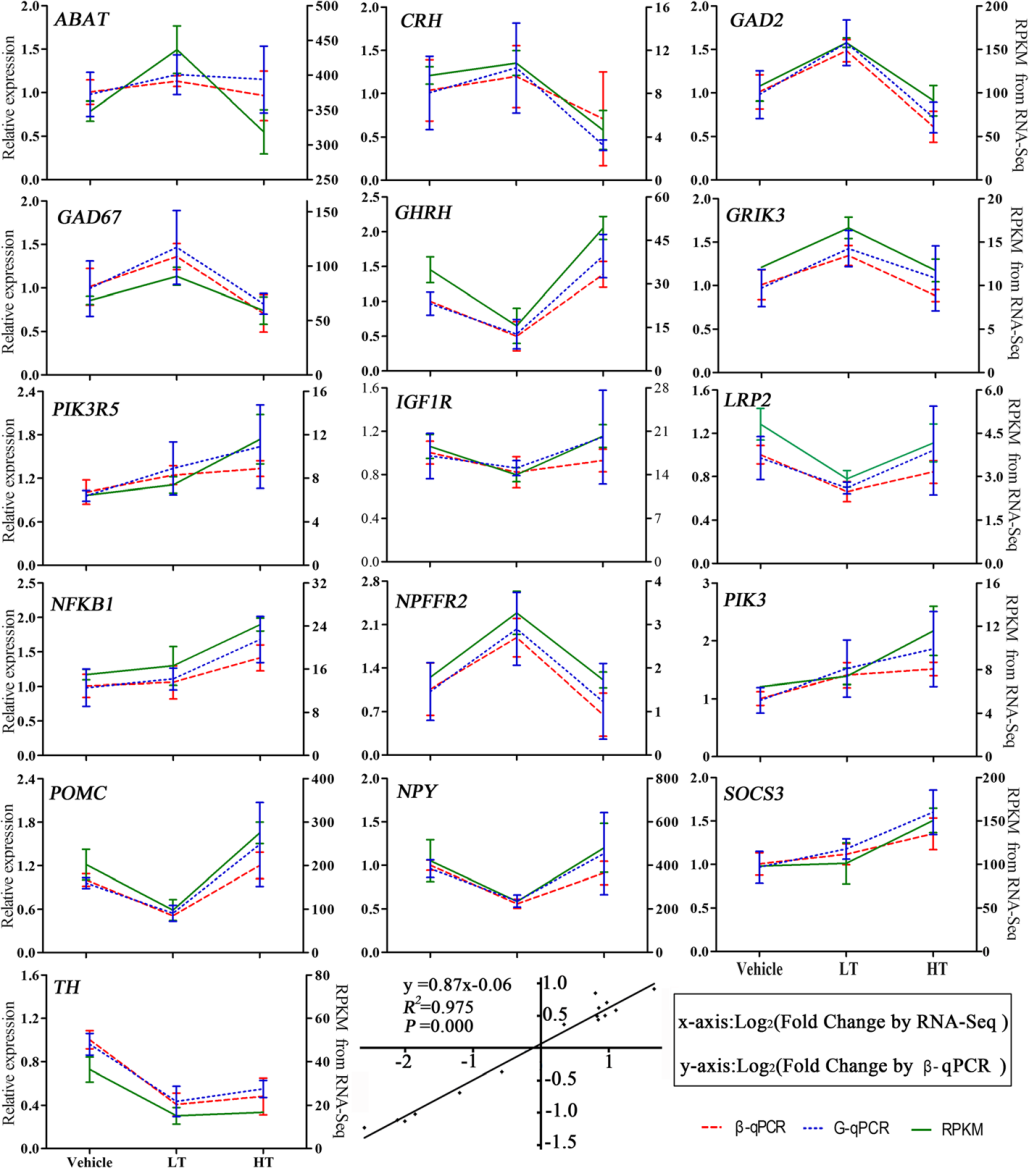
Gene expression levels measured by qRT-PCR were compared with those measured by RNA-seq. The left axis represents gene expression levels determined by qRT-PCR, and the right axis represents expression levels determined by RNA-seq in RPKM units. Each dot represents the relative expression data for a given gene and a given sample. Bars represent the mean (± SE) of three experiments

To confirm the differential expression values obtained from the statistical comparison of sequencing data, qRT-PCR was performed to validate the DEGs involved in the regulation of feed intake. A total of 16 DEGs of 7 types were selected, including NPFFR2, ABAT, GRIK3, GAD67 and GAD2 from cluster 3; NPY and NFKB1 from cluster 4; CRH from cluster 5; POMC, GHRH and IGF1R from cluster 6; LRP2 and TH from cluster 7; and PIK3, SOCS3 and PIK3R5 in the insulin signaling pathway and adipocytokine signaling pathways. We found highly significant and positive correlations, and the correlation coefficient was 0.975 (*P* < 0.01) between the qRT-PCR and RNA-Seq results according to Villacorta-Martín et al. and Yu et al. [37, 38]. Furthermore, the correlation between relative expression (β-actin as the reference gene) and expression (GAPDH as the reference gene) was assessed by Pearson’s correlation analysis using SPSS software. The correlation coefficient was 0.949 (*P* = 0.000). Therefore, the expression patterns of these 16 DEGs were consistent with the RNA-seq results (Fig. 9).

## Discussion

The hypothalamus is widely regarded as the center regulating feed intake in animals [39]. Many neural peptides with suppressive and promotive effects on food consumption have been identified in mammals, and similar functions have also been validated in poultry. Studies have shown the key role of visfatin in the regulation of poultry feed intake, glucose homeostasis and energy balance, but the mechanism remains unclear. The current study analyzed the effects of visfatin on chick feed intake and related blood biochemical indexes as well as the associated molecular mechanism using ICV injection of recombinant human visfatin into Roman Brown cockerels. Compared with that in the C group, feed intake was significantly increased in chicks at 60 min post-injection in the LT and HD groups, which was consistent with the results of Cline et al. [29]. In addition, Cline et al. found that the amount of feed intake in chicks was significantly higher in the HT group (0.25 nmol) than in the LT group (0.025 nmol). In our study, the feed intake was not significantly different between the HT group and the LT group (*P* > 0.05), suggesting that the feed intake was not dependent on the dose of visfatin. Similar results have been found in rats; these results showed that visfatin significantly increased food intake at 2 and 24 h post-injection [30].

Plasma metabolites can reflect the production performance and nutrition level of poultry to some extent, allowing their nutrition to be regulated in a timely manner. Brown et al. [40] found that visfatin significantly induced insulin secretion in rats and affected the phosphorylation of the insulin receptor and the expression of a series of islet β cell function-related genes. Visfatin participates in the regulation of glucose metabolism by binding with the insulin receptor [20], thus reducing the secretion of endogenous insulin via negative feedback signaling to the central nervous system. Our study found a significant decrease in the serum TG level in Roman Brown cockerels after the ICV injection of visfatin, which was inconsistent with the reports in mammals. Zeynep et al. reported that the visfatin protein concentrations were negatively correlated with blood HDL and cholesterol levels [41]. This shows that visfatin not only participates in the metabolism of TGs but also plays an important role in lipid metabolism. In our study, feed intake and the concentration of insulin, TGs, cholesterol and LDL were significantly changed at 60 min after ICV injection. Therefore, visfatin is involved in the regulation of feed intake and lipid metabolism [29, 30].

Analyses revealed 4 common DEGs among the LT, HT and C groups, including *TH*, *GHRH* and two new genes. On the one hand, TH and GHRH might play important roles in the functions of visfatin, and the two new genes are likely to have similar functions as *TH* and *GHRH*; on the other hand, the different pathways of activity of high and low dose of visfatin on feed intake may have altered the expression of these genes. *TH* is the rate-limiting enzyme in the synthesis of catecholamines, including DA [42]. The hypothalamic-pituitary-adrenal (HPA) axis and DA can interact with each other [43]. The activation of the HPA axis in animals is caused by the release of opioids. It is well known that endogenous opioids and systemic opiate agonists can increase food intake, while opiate antagonists have the opposite effect [44]. Brunetti et al. [30, 31] demonstrated that visfatin promotes appetite in rats, and this effect may be mediated by the activity of DA and CRH in the hypothalamus and the reduction in the expression of corresponding genes. Based on the direction of change, TH was assigned to cluster 7, which was down-regulated in the LT and HT groups. TH reduced DA concentration in the hypothalamus and promoted chick feeding, which was consistent with the results of Brunetti et al. [30]. The major function of GHRH in the hypothalamus is regulating the release of growth hormone (GH), and there is a close correlation between GH concentration and the growth rate in animals. In a previous study, GHRH was identified as a suppressive factor for poultry feed intake [3]. However, in this study, the expression of GHRH was significantly reduced in the LT group compared to that in the C group, which promoted feeding in chicks. It can be speculated that visfatin directly or indirectly regulated TH and GHRH expression to regulate feed intake in chicks. Interestingly, the two novel genes in cluster 1 were significantly up-regulated between the C and LT and between the LT and HT comparative groups. Therefore, the specific mechanism of these two genes, which may be important in feeding regulation induced by visfatin, deserves further study.

Cluster analysis showed the presence of several appetite-associated DEGs. The expression of DEGs was significantly increased in clusters 1, 2 and 4, including *HCRT* [45]*, GnRH-I* [46], *NPY, AgRP*, *TSHB*, *CGA*, *SST*, *CHRNA4* and *PDYN*. In chickens, GnRH-I is mainly distributed in the hypothalamus and regulates pituitary gonadotropin release [47]. The ad libitum feeding of chronically food-restricted laying broiler hens for 7 days increased hypothalamic GnRH-I mRNA levels [46]. Thus, the discrepancy in the effects of GnRH-I between chicks and mammals might be related to the differences in the appetite regulatory mechanisms between these two classes of animals. In the hypothalamus of avian species, 2 primary populations of neurons influence appetite through the release of signaling molecules: orexigenic neuropeptides (NPY/AgRP) [48] and anorexigenic neuropeptides (POMC/CRH) [49–51]. NPY can promote increased feed intake in both mammals, such as rats, and in poultry [52]. In a hungry or food-restricted state, the transcription of NPY was significantly enhanced in the hypothalamus of broilers, and the activity of neurons was also increased. Additionally, the ICV injection of NPY could significantly promote feeding behavior [53]. AgRP is a natural antagonist of melanocortin, which competes with α-MSH, an agonist of melanocortin, to bind to MC4R and inhibit its anorexic effects [54]. Systemic injection of AgRP can increase feeding behavior, and this effect lasts longer than the feeding effect increased by the systemic injection of NPY [55]. The ICV injection of AgRP can promote ad libitum feed intake in egg-laying hens [56]. In our study, the expression of NPY and AgRP increased as the dose of visfatin increased. Thus, these genes were grouped into cluster 4. Visfatin expression has been reported to be correlated with NPY expression in adipose tissue, and positively correlated with NPYR 1, 2, and 5 in visceral fat tissues [20, 57]. Therefore, visfatin may directly or indirectly regulate NPY and AgRP to promote feed intake in chicks.

SST is involved in the control of food intake. It can directly suppress POMC mRNA expression and ACTH release in primary mouse pituitary cells [58], thereby strongly supporting the hypothesis that endogenous SST can act as a corticotropin synthesis-inhibiting factor [59]. POMC is synthesized in the hypothalamic arcuate nucleus and antehypophysis. ACTH is a peptide derived from POMC, and α-MSH can be derived from the first 13 amino acids of ACTH [60]. α-MSH, which is derived from a multifunctional precursor protein, proopiomelanocortin, plays an inhibitory role in food intake [61]. POMC, a precursor of α-MSH, lipotropins and β-endorphin, is one of the important factors in the hypothalamus suppressing appetite [62], and α-MSH, as a melanocortin agonist, can act on melanocortin receptors MC3R and MC4R in the poultry hypothalamus as an anorexia signal, thereby suppressing appetite and promoting energy consumption [56, 63]. CRH is the most important regulatory factor of the HPA axis, playing an important role in feed intake and energy balance. Lateral ventricle injection of CRH can significantly reduce feed intake of chicks, demonstrating the suppressive effect of CRH on appetite via the central nervous system [64]. Brunetti et al. increased feed intake in rats via the ICV injection of visfatin, and this was likely mediated by the decrease in DA and CRH expression in the hypothalamus [44]. These results suggest that in the hypothalamus, visfatin may be involved in the expression of NPY/AgRP and POMC/CRH genes related to feeding behavior.

CRH, TRH and AVP synthesized and secreted by the hypothalamus can not only alter the secretion of pituitary gland, thyroid and adrenal hormones but also play an important role in regulation of many physiological processes. In our study, compared with the C group, AVP and TRH were significantly up-regulated in the LT group, which was not different in the HT group, while TRHR expression was down-regulated. Additionally, CRH and TRHR expression was down-regulated in cluster 5, which was consistent with previous reports [65–67]. CGA plays an autocrine role as a glucocorticoid-responsive inhibitor of POMC-derived peptide secretion [68]. In our study, CGA was up-regulated, while POMC was down-regulated. Therefore, it is likely that CGA inhibits POMC, thus promoting feed intake in chicks. Thyrotrophs synthesize and secrete TSH, a hormone composed of two subunits, namely, CGA and TSHB, which confer the biological hormonal activity to TSH and are rate limiting in the formation of mature TSH [69, 70]. In our study, CGA and TSHB were persistently up-regulated. The physiological function of TSH is to bind with TSHR located on thyroid follicular cells to activate cAMP signaling pathways, thus promoting the synthesis and release of THs. TSH indirectly affects the glucolipid metabolism by regulating the synthesis and release of THs [71].

There were other DEGs related to feed intake, such as CCK, GLP-1R, PMCH, GHRH, GHR, GnIH, BMP7 and CNR1 [72–81]. CCK, a polypeptide hormone secreted by gastrointestinal mucosa cells, causes the gallbladder to shrink, promotes the secretion of pancreatic enzymes and participates in the regulation of animal feed intake. Exogenous CCK injection can reduce feed intake in chicks [72]. CCK binds to its receptor to activate POMC neurons and causes appetite suppression via satiation signals sent by its receptor MC4R [73]. GLP-1R is the receptor of GLP-1, which is an incretin hormone that can significantly inhibit feed intake in broilers at a low dose [52]. GLP-1R activation reduces food intake, and, conversely, a decrease in GLP-1R activity increases food intake [75]. In our study, the expression of CCK and GLP-1R was significantly lower in the LT and HT groups than that of the C group, and the results were consistent with those of others [52, 72–76]. GnIH and CNR1 can promote feed intake in chicks [77–80]. ICV administration of GnIH is associated with increased food intake via opioid receptors in chicks [77, 78]. CNR1 also up-regulates orexigenic NPY and MCH in the lateral hypothalamus [79, 80]. In our study, we found a significant increase in hypothalamic GnIH expression after visfatin treatment, which could be related to the feeding-promotive role of visfatin. BMP7 decreases food intake [81], and it was down-regulated in our study.

KEGG pathway analysis demonstrated that the neuroactive ligand-receptor interaction pathway was most significantly affected by visfatin. We found that 30 DEGs were enriched in the neuroactive ligand-receptor interaction pathway, including the appetite-related genes VIP, NPY, AgRP, POMC, CRH, TRH, CGA, TSHB and TH. Most of these genes are receptor genes associated with feed intake regulation; in addition, there were many ligand genes associated with feed intake regulation (Fig. 7). Interestingly, among the three groups, there were significant correlations between the changes in the expression of receptor genes and ligand genes (Additional file 7: Table S5). These results indicate that the ICV injection of visfatin increased feed intake partially by regulating the neuroactive ligand-receptor interaction pathway. After ICV injection of visfatin, genes associated with feed intake regulation were expressed, and the ligands bound to the receptors to increase feed intake in chicks. Thus, the neuroactive ligand-receptor interaction pathway plays an important role in the regulation of feed intake after ICV injection of visfatin in chicks.

An increase in AMPK can activate both ACC and TSC pathways by increasing the phosphorylation of the respective factors. Reduced activity of ACC prevents the formation of malonyl-CoA and, by promoting the mitochondrial enzyme CPT1, leads to a decrease in the cellular levels of ACSL1. ACSL1 decreases the expression of AgRP and NPY and suppresses feed intake [82]. Alternatively, an increase in AMPK can inhibit the activity of mTOR by increasing the phosphorylation of mTOR or TSC [83]. The activation of the mTOR pathway can regulate AgRP/NPY neurons through P70s6k or the translation initiation factor 4EBP1 [84, 85]. However, these genes were not significantly altered among the three groups in our study. These results show that visfatin regulates feeding behavior by pathways other than the AMPK-ACC-CPT1-NPY and AMPK-mTOR-p70s6k/4EBP1-NPY pathways.

In our study, several inflammation- and immune-related genes, including IL-8, IL-13, IL-22, IL-34, NFKB1, CD83 and CD44, were also found to be differentially expressed. Recent studies have suggested that visfatin may have a role in the regulation of peripheral inflammatory responses [22]. Visfatin was shown to activate NFKB1 [15]. Visfatin can induce the secretion of the proinflammatory cytokines IL-1β and IL-6 [86]. Therefore, these genes can be induced by visfatin in chicks.

We mapped the DEGs onto PPI networks and constructed the signal transduction and transcriptional regulatory network. In the LT vs HT groups, 8 DEGs including VIP, NPY, AgRP, POMC, CRH, TRH, CGA, TSHB and TH were enriched in the appetite-related interaction network. In the C vs LT groups, 6 DEGs including POMC, SST, DDC, VIP and TH were enriched in the appetite-related interaction network. POMC, an anorexigenic neuropeptide, was the hub gene that interacted with the maximum number of nodes. Therefore, these genes and pathways may play important roles in appetite regulation after the ICV injection of visfatin [62]. The orexigenic effects of visfatin may be mediated by its role in the synthesis of POMC and α-MSH [31]. Hence, visfatin induces feeding through the inhibitor of the hypothalamic POMC/CRH.

## Conclusion

In conclusion, ICV visfatin exerts orexigenic effects on chicks. The results described in the present study suggest that visfatin might have important functions in regulating feed intake, and these functions might be mediated by the visfatin-POMC-CRH signaling pathway. We also demonstrated that visfatin induces hyperphagia in chicks through the activation of hypothalamic signals partially by regulating the neuroactive ligand-receptor interaction pathway. Therefore, we provide the first direct evidence that visfatin stimulates the central nervous system to influence food intake in chicks.

## Methods

### Animals and ICV injection

The experiments and animal care were performed according to the Regulations for the Administration of Affairs Concerning Experimental Animals (Ministry of Science and Technology, China, 2004) and were approved by the Institutional Animal Care and Use Committee (Use Committee of Henan Agricultural University, China; Permit Number: 17-0118). All of the experiments and methods were performed in accordance with approved guidelines. On the morning of hatch, egg-type cockerels were obtained from a commercial hatchery. They were maintained in accordance with hatchery recommendations and were given free access to water and a commercial diet. At 10 days of age, chicks of similar body weights (100 ± 5 g) were anesthetized by iv injection in the wing vein with pentobarbital sodium, and the ICV injection was performed according to a method adapted from Davis et al. [87]. Briefly, the head of each chick was placed inside an acrylic box that had a hole on the top. The drug solution was injected through the hole, with a microsyringe. The injection coordinates were 3 mm anterior to the coronal suture, 1 mm lateral from the sagittal suture, and 2 mm deep targeting the left lateral ventricle. Human recombinant visfatin (BioVision, Mountain View, CA, USA) was dissolved in artificial cerebrospinal fluid to constitute a total injection volume of 5 μl with 0.1% Evans Blue dye to facilitate the identification of the injection site. All of the injection drugs and vehicle contained 0.1% Evans blue solution to facilitate injection site localization.

### Determination of feed intake and plasma metabolites

After the hypothalamus catheter was embedded, the chicks were allowed to recover for 3 days before experimental injection [88]. Seventy-two 13-day-old Roman Brown cockerels were randomly divided into three treatment groups. Each treatment group had 3 replicate groups including 8 chicks. The chicks were fasted for 3 h and then randomly intracerebroventricularly injected with 0 ng (vehicle only, C), 40 ng (low dose treatment, LT) and 400 ng (high dose treatment, HT) visfatin. The food intake was measured, and blood samples were drawn from the wing vein every 30 min for 90 min after injection. The food scattered under the feeders was collected, weighed, and deducted from food intake. Biochemical indexes in the serum, including glucose, triglyceride (TG), total cholesterol, high-density lipoprotein (HDL) and low-density lipoprotein (LDL) levels, were measured with an automatic blood biochemical analyzer, and the level of insulin was tested using an ELISA kit.

### Total RNA isolation and RNA-seq

Each chick was randomly assigned to receive vehicle, 40 ng or 400 ng visfatin by ICV injection. At 60 min post-injection, the chicks were deeply anesthetized with sodium pentobarbital, and sacrificed via cardiopuncture; their brains were removed, and the whole hypothalamus was collected in liquid nitrogen. A total of 9 samples were successfully collected. Using RNAiso Plus reagent (TaKaRa, Dalian, China), total RNA was extracted from the pectoral hypothalamus of Roman Brown cockerels and used in library construction; 3 biological replicates were performed. RNA degradation and contamination were assessed with 1% agarose gels. The purity, concentration and integrity of RNA were checked using the NanoPhotometer spectrophotometer, Qubit RNA Assay Kit and Qubit 2.0 Fluorometer, and RNA Nano 6000 Assay Kit and Bioanalyzer 2100, respectively. Sequencing libraries were generated using NEBNext Ultra Directional RNA Library Prep Kit for Illumina (NEB, USA) following the manufacturer’s recommendations, and index codes were added to attribute sequences to each sample. The libraries were sequenced on an Illumina HiSeqXTen by Novogene Biotech Co., Ltd. The sequencing data were submitted to the NCBI Sequence Read Archive under Accession SRP112390. Raw data in the FASTQ format were first processed through in-house Perl scripts. Additionally, the quality score of 30 (Q30) and GC content of the cleaned data were calculated. All downstream analyses were based on high-quality clean data.

### Read mapping and quantification of gene expression level

Reads that passed the quality control test were aligned to the *Gallus gallus* reference genome (Gal5) from NCBI using TopHat (version 2.0.9). According to the alignment results from TopHat, the transcriptome data for each library were assembled separately using Cufflinks (version 2.1.1). HTSeq (version 0.6.1) was used to count the read numbers mapped to each gene. The transcript abundances are shown as reads per kilobase of exon model per million mapped reads (RPKM). For differential expression analysis, gene expression levels of three groups were analyzed using DESeq. The differentially expressed genes (DEGs) between each pair of samples were screened using a model based on the negative binomial distribution. Multiple corrections for the *P*-value were performed using the Benjamini-Hochberg’s approach for controlling the false discovery rate. Genes with an adjusted *P*-value < 0.05 identified by DESeq were labeled differentially expressed. To gain insights into the DEGs specifically induced by visfatin at each time point, analyses were performed between C and LT, C and HT, and LT and HT groups.

### Series-cluster analysis

Based on the trend in expression, series-cluster analysis of these DEGs classified them into eight clusters among the three groups. However, the genes were only significantly enriched in eight clusters: significantly up-regulated DEGs in both C vs LT and LT vs HT were classified as cluster 1; significantly up-regulated DEGs in C vs LT and non-significantly changed in LT vs HT were classified as cluster 2; significantly up-regulated DEGs in C vs LT and significantly down-regulated in LT vs HT were classified as cluster 3; non-significantly changed DEGs in C vs LT and significantly up-regulated in LT vs HT were classified as cluster 4; non-significantly changed in C vs LT and significantly down-regulated DEGs in LT vs HT were classified as cluster 5, significantly down-regulated DEGs in C vs LT and significantly up-regulated in LT vs HT were classified as cluster 6; significantly down-regulated DEGs in C vs LT and non-significantly changed in LT vs HT were classified as cluster 7; and significantly down-regulated DEGs in both C vs LT and LT vs HT were classified as cluster 8.

### GO, KEGG enrichment analysis of DEGs

GO enrichment analysis of DEGs was performed using the GOseq package in R, in which the gene length bias was corrected. GO terms with corrected *P*-values < 0.05 were considered significantly enriched by the DEGs. Kyoto Encyclopedia for Genes and Genomes (KEGG) is a database for the elucidation of high-level functions of biological systems, such as at the level of the cell, the organism and the ecosystem, using molecular-level information (http://www.genome.jp/kegg/). We used KOBAS software to test the statistical enrichment of the DEGs in KEGG pathways.

### PPI analysis of DEGs

Protein-protein interaction (PPI) analysis of DEGs was based on the STRING database. We constructed PPI networks by extracting the target gene list from the database pertaining to chickens. In this study, the online tool STRING was applied to analyze the PPI of DEGs, and PPI with scores > 700 were selected as significant interactions.

### Validation of DEGs by qRT-PCR

To confirm the reproducibility and accuracy of the RNA-seq data, a total of 16 DEGs of 7 types were selected for quantitative real-time PCR (qRT-PCR) analysis. Then, first-strand cDNA was synthesized using the PrimeScript RT Reagent Kit with gDNA Eraser according to the manufacturer’s instructions (TaKaRa, Dalian, China). The qRT-PCR was performed in a 25 μL reaction volume containing 100 ng of cDNA, 12.5 μL of 2× SYBR® Premix Ex Taq™ II (TaKaRa, Dalian, China), 10 μM each of the primers and deionized water on a LightCycler® 96 Real-Time PCR system (Roche Applied Science, Indianapolis, USA). The relative gene expression levels were calculated with the comparative CT method (also referred to as the 2^-△△CT^ method) using β-actin and GAPDH as the reference genes. The qRT-PCR amplification procedure was as follows: 95 °C for 3 min; 40 cycles at 95 °C for 12 s, 61 °C for 40 s, and at 72 °C for 30 s; and extension at 72 °C for 10 min. The primers are shown in Additional file 8: Table S1.

### Data analysis

Statistical analyses of the data were performed using SPSS version 24.0 for Windows (SPSS Inc., Chicago, IL, USA). The data are presented as the mean ± SE. Intergroup differences were compared using one-way ANOVA for continuous variables for determination of feed intake and plasma metabolites. *P* < 0.05 was considered statistically significant.

Statistical analyses of the data were performed using SPSS version 24.0 for Windows (SPSS Inc., Chicago, IL, USA). Intergroup differences were compared using one-way ANOVA for continuous variables for determination of feed intake and plasma metabolites. The data are presented as the mean ± SE. *P* < 0.05 was considered statistically significant. Correlation analysis of RNA-Seq and q-PCR data from selected genes was performed using a linear regression model. The linear regression model was as follows: *y*_*i*_ = a*x*_*i*_ + b, where *y*_*i*_ represents the q-PCR value, log_2_ (fold change by β-qPCR), at the *i*th gene; and *x*_*i*_ represents the RNA-seq value, log_2_ (fold change by RNA-Seq), at the *i*th gene.

## Additional files

Additional file 1: **Figure S1.** Pearson correlation between samples. (TIFF 287 kb)

Additional file 2: **Table S2.** DEGs in the three comparisons. (XLSX 102 kb)

Additional file 3: **Table S3.** GO analyses of DEGs in the three comparisons. (XLSX 328 kb)

Additional file 4: **Figure S2.** The top 30 most significantly enriched GO terms in the three comparisons (LT vs C, HT vs C, and LT vs HT). (TIFF 1661 kb)

Additional file 5: **Figure S3.** Analyses of KEGG pathway enrichment. The x-axis shows the enrichment factor; the y-axis corresponds to the KEGG pathway. The color of the dot represents the q value, and the size of the dot represents the number of DEGs mapped to the reference pathways. A, B and C represent the top 20 KEGG pathways enriched for the DEGs observed in the LT vs C, HT vs C, and LT vs HT groups. (TIFF 1178 kb)

Additional file 6: **Table S4.** KEGG analyses of DEGs in the three comparisons. (XLSX 31 kb)

Additional file 7: **Table S5.** The correlation coefficient between receptor genes expression and ligand genes expression in the neuroactive-ligand receptor interaction pathway. (XLSX 15 kb)

Additional file 8: **Table S1.** List of the genes and primers used for qRT-PCR validation. (DOCX 17 kb)

## Abbreviations

4EBP1: 4E-binding protein
ABAT: 4-aminobutyrate aminotransferase
ABCA4: ATP-binding cassette subfamily A member 4
AgRP: Agouti-related protein
AVP: Arginine vasopressin
BMP7: Bone morphogenetic protein 7
CCK: Cholecystokinin
CGA: Common glycoprotein alpha chain
CHRNA4: Cholinergic receptor nicotinic alpha 4 subunit
CNP3: Natriuretic peptide C type 3
CNR1: Cannabinoid receptor 1
CPT1: Carnitine palmitoyltransferase-1
CRF: Corticotropin releasing factor
CRH: Corticotropin releasing hormone
DA: Dopamine
GAD67: Glutamate decarboxylase 1, 67 kDa
GAL: Galanin
GALP: Galanin-like peptide
GHR: Growth hormone receptor
GHRH: Growth hormone-releasing hormone
GLP-1R: Glucagon-like peptide 1 receptor
GnIH: Gonadotropin-inhibitory hormone
GnRH-I: Gonadotropin-releasing hormone I
HCRT: Hypocretin neuropeptide precursor
ICV: Intracerebroventricular
IGF1R: Insulin-like growth factor 1 receptor
LRP2: LDL receptor-related protein 2
MCH: Melanin-concentrating hormone
MST: Mesotocin-neurophysin I
mTOR: Mammalian target of rapamycin
NFKB1: Nuclear factor kappa B subunit 1
NFKBIZ: Nuclear factor kappa B inhibitor zeta
NMBR: Neuromedin B receptor
NPBWR1: Neuropeptides B/W receptor 1
NPFFR2: Neuropeptide FF receptor 2
NPY: Neuropeptide Y
ORX: Orexin
P70s6k: P70 ribosomal S6 kinase 1
PDYN: Prodynorphin
PIK3R5: Phosphoinositide 3-kinase regulatory subunit 5
PMCH: Pre-pro melanin-concentrating hormone
POMC: Proopiomelanocortin
PTGFR: Prostaglandin F receptor
PTH-L: Parathyroid hormone-like
PYY: Peptide YY
SHH: Sonic hedgehog
SOCS1: Suppressor of cytokine signaling 1
SST: somatostatin
TH: Thyroid hormone
TRH: Thyrotropin-releasing hormone
TRHR: Thyrotropin-releasing hormone receptor
TSC: Tuberous sclerosis complex
TSHB: Thyroid-stimulating hormone beta
TSHR: Thyroid-stimulating hormone receptor
VIP: Vasoactive intestinal peptide
