# Supplementary figures and images for: Comparative transcriptome analysis of hypothalamus-regulated feed intake induced by exogenous visfatin in chicks
Source: BMC Genomics. 2018 Apr 11;19:249. doi: 10.1186/s12864-018-4644-7 (PMC5896085; doi:10.1186/s12864-018-4644-7)

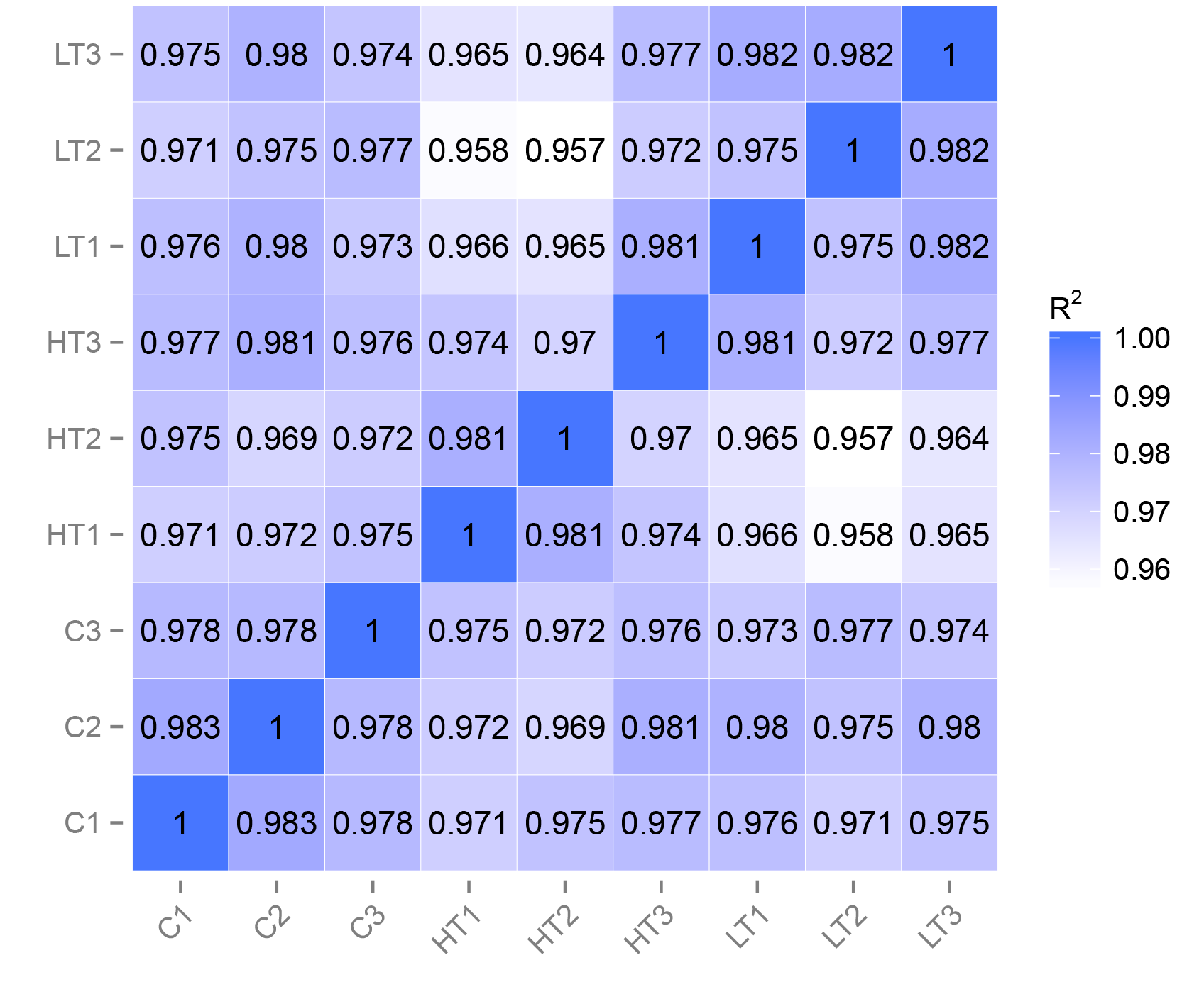

Supplement: Supplementary file 1 — Figure S1. Pearson correlation between samples. (TIFF 287 kb) [file 12864_2018_4644_MOESM1_ESM.tif]

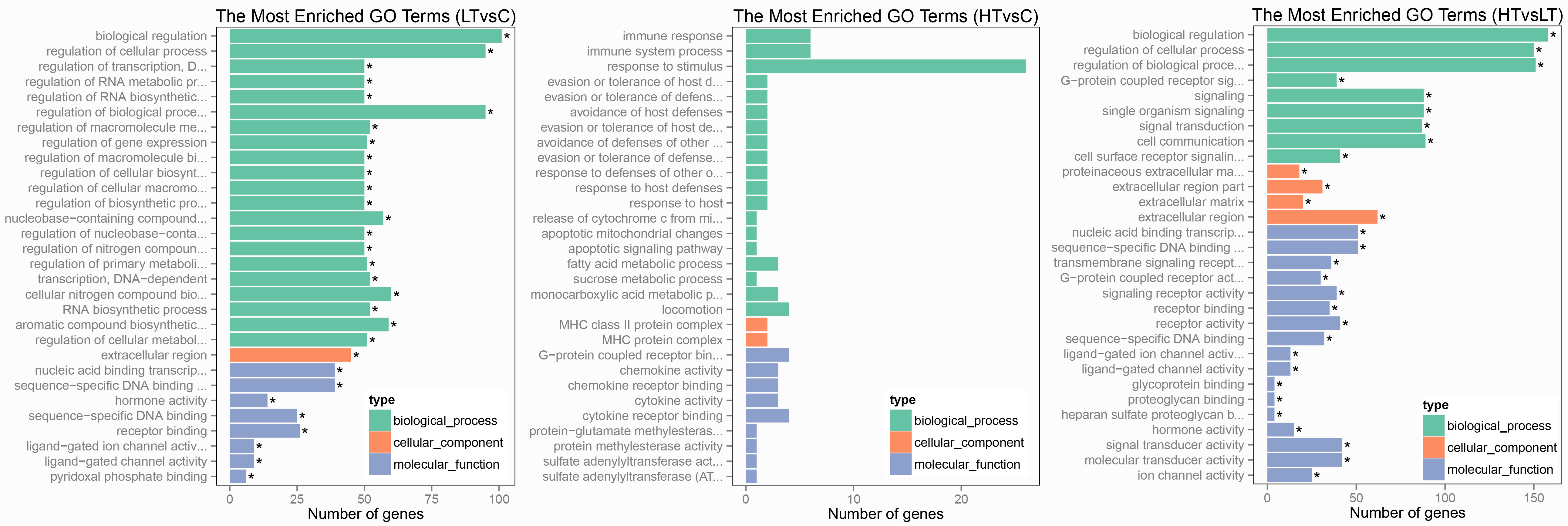

Supplement: Supplementary file 4 — Figure S2. The top 30 most significantly enriched GO terms in the three comparisons (LT vs C, HT vs C, and LT vs HT). (TIFF 1661 kb) [file 12864_2018_4644_MOESM4_ESM.tif]

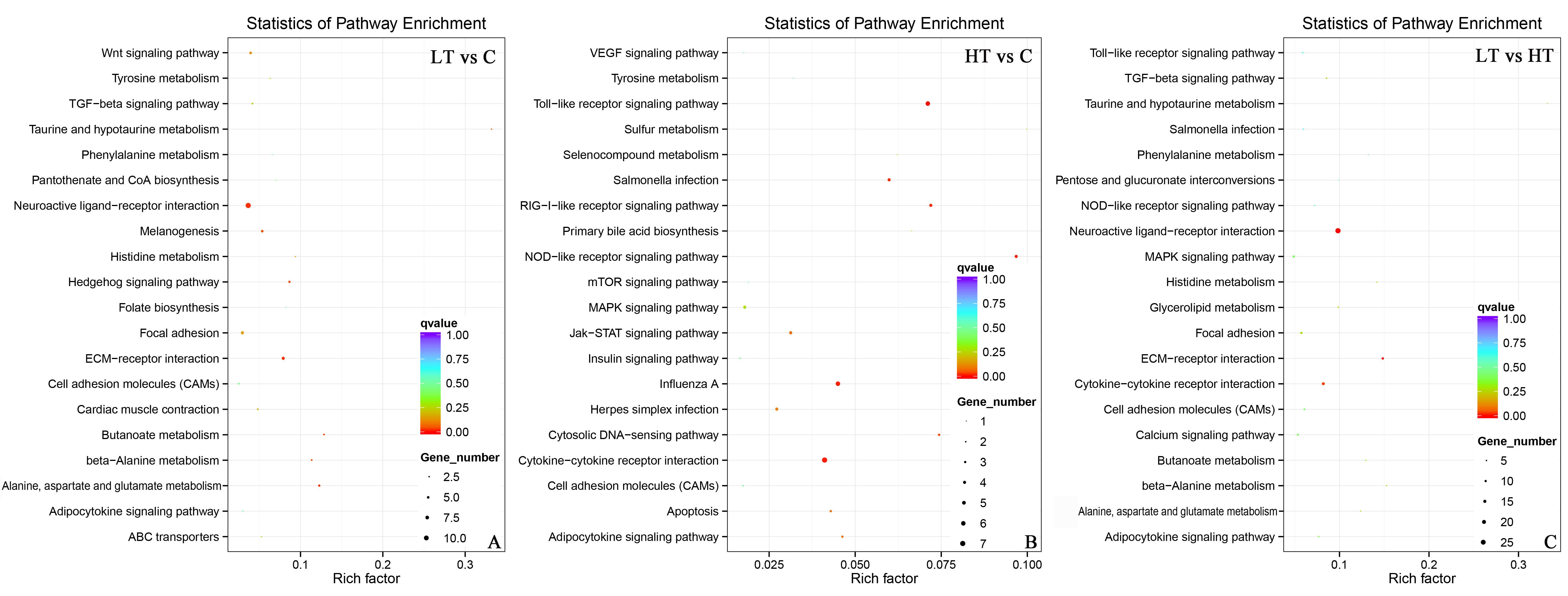

Supplement: Supplementary file 5 — Figure S3. Analyses of KEGG pathway enrichment. The x-axis shows the enrichment factor; the y-axis corresponds to the KEGG pathway. The color of the dot represents the q value, and the size of the dot represents the number of DEGs mapped to the reference pathways. A, B and C represent the top 20 KEGG pathways enriched for the DEGs observed in the LT vs C, HT vs C, and LT vs HT groups. (TIFF 1178 kb) [file 12864_2018_4644_MOESM5_ESM.tif]
