# Supplementary material for: Comparative transcriptome analysis of hypothalamus-regulated feed intake induced by exogenous visfatin in chicks
Source: BMC Genomics. 2018 Apr 11;19:249. doi: 10.1186/s12864-018-4644-7 (PMC5896085; doi:10.1186/s12864-018-4644-7)
Supplement: Supplementary file 8 — Table S1. List of the genes and primers used for qRT-PCR validation. (DOCX 17 kb) [file 12864_2018_4644_MOESM8_ESM.docx]

| **Gene** | **Accession ID** | **Sequences(5’-3’)(forward/reverse）** | **Amplicon**  **size (bp)** |
| --- | --- | --- | --- |
| TH | NM_204805.2 | GACCAGAACTACCAGCCTGT/GTGGGTGTAGGGCTCGTATT | 123 |
| GHRH | XM_015296359.1 | AGCTCCATCTCCTCTCCTCT/AAACCCTCCTCGTCCTGTTC | 143 |
| POMC | XM_015285103.1 | AGGGTTGGAACGAGAGGAAG/ACGAGCCTCCATCCTTCTTT | 248 |
| GAD2 | XM_015282054.1 | GCGGCATGGAAGACAATGAA/ATGAGTTGCTGCTGGGTTTG | 164 |
| GRIK3 | XM_417766.4 | CTTCCCTTAGCCATGCCATC/CGAAGATGATGCGGAACTCC | 225 |
| ABAT | XM_004945322.2 | CAGGCTGCTCGTATTTGGAC/CCCTCGGCTCTCTTCGTAAT | 193 |
| SOCS3 | NM_204600.1 | ACCACTACATGCCTCCCAC/GACAGTCTTACGGCAGAGGT | 170 |
| NFKB1 | XM_015285415.1 | CTCCTCAAGCATCAGAAGGC/CATCTGCATCACCCTCAAGC | 235 |
| PIK3R5 | NM_001030697.1 | CTGCCCTACACTGACAAGG/GCCGATGTAGTGGGAGATGT | 196 |
| IGF1R | NM_205032.1 | CGGTATCGCCCTCCAGATTA/CCAAGGTTTCAATCCCTGCA | 192 |
| NPFFR2 | NM_001034825.2 | TACGGTTCTGAGGAGCAAGC/TGGAGGCAGAGACAGAGATTC | 194 |
| LRP2 | XM_004942763.2 | ACGTACAGACACACCTCAGG/AGGAGAGGAAGGAGGGACAT | 142 |
| CRH | NM_001123031.1 | GAAGCCTCCGCCGAGTTG/CAGCAGGTGGAAAGTCAGGT | 150 |
| NPY | NM_205473.1 | CGGCTCTGAGGCACTACATC/TCCCATCACCACATCGAAGG | 151 |
| GAD67 | NM_204913.1 | CTCGCATTAAAGTTCCGCCG/CTCGGAGTTCGCCTTTCCTT | 193 |
| PIK3 | XM_015288344.1 | CTGCACCAACATGGGGTTTC/GTACACGACACCTGATCCTGC | 183 |
| β-actin | NM_205518.1 | AGTACCCCATTGAACACGGT/ATACATGGCTGGGGTGTTGA | 197 |
| GAPDH | NM_204305.1 | AGAACATCATCCCAGCGT/AGCCTTCACTACCCTCTTG | 184 |

Table S1 List of the genes and primers used for qRT-PCR validation
